# How equitable is the uptake of conditional cash transfers for maternity care in India? Evidence from the Janani Suraksha Yojana scheme in Odisha and Jharkhand

**DOI:** 10.1186/s12939-017-0539-5

**Published:** 2017-03-10

**Authors:** Nattawut Thongkong, Ellen van de Poel, Swati Sarbani Roy, Shibanand Rath, Tanja A. J. Houweling

**Affiliations:** 1 Institute of Health Policy and Management (iBMG), Erasmus University Rotterdam, Rotterdam, The Netherlands; 2 Ekjut, Plot 556B, Potka, Chakradharpur, West Singhbhum, Jharkhand PIN: 833102 India; 3 Department of Public Health, Erasmus MC Rotterdam, P.O. Box 2040, 3000 CA Rotterdam, The Netherlands

**Keywords:** Socioeconomic inequality, Conditional cash transfers, Maternity care, Janani Suraksha Yojana, India

## Abstract

**Background:**

In 2005, the Indian Government introduced the Janani Suraksha Yojana (JSY) scheme - a conditional cash transfer program that incentivizes women to deliver in a health facility – in order to reduce maternal and neonatal mortality. Our study aimed to measure and explain socioeconomic inequality in the receipt of JSY benefits.

**Methods:**

We used prospectively collected data on 3,682 births (in 2009–2010) from a demographic surveillance system in five districts in Jharkhand and Odisha state, India. Linear probability models were used to identify the determinants of receipt of JSY benefits. Poor-rich inequality in the receipt of JSY benefits was measured by a corrected concentration index (CI), and the most important drivers of this inequality were identified using decomposition techniques.

**Results:**

While the majority of women had heard of the scheme (94% in Odisha, 85% in Jharkhand), receipt of JSY benefits was comparatively low (62% in Odisha, 20% in Jharkhand). Receipt of the benefits was highly variable by district, especially in Jharkhand, where 5% of women in Godda district received the benefits, compared with 40% of women in Ranchi district. There were substantial pro-rich inequalities in JSY receipt (CI 0.10, standard deviation (SD) 0.03 in Odisha; CI 0.18, SD 0.02 in Jharkhand) and in the institutional delivery rate (CI 0.16, SD 0.03 in Odisha; CI 0.30, SD 0.02 in Jharkhand). Delivery in a public facility was an important determinant of receipt of JSY benefits and explained a substantial part of the observed poor-rich inequalities in receipt of the benefits. Yet, even among public facility births in Jharkhand, pro-rich inequality in JSY receipt was substantial (CI 0.14, SD 0.05). This was largely explained by district-level differences in wealth and JSY receipt. Conversely, in Odisha, poorer women delivering in a government institution were at least as likely to receive JSY benefits as richer women (CI −0.05, SD 0.03).

**Conclusion:**

JSY benefits were not equally distributed, favouring wealthier groups. These inequalities in turn reflected pro-rich inequalities in the institutional delivery. The JSY scheme is currently not sufficient to close the poor-rich gap in institutional delivery rate. Important barriers to institutional delivery remain to be addressed and more support is needed for low performing districts and states.

## Background

Universal Health Coverage, defined as ensuring that everyone has access to affordable and quality health care, features high on the policy agenda of many low and middle income countries. While the focus on maternal and child health (MCH) in the Millennium Development Goals (MDG) has brought about much progress in the coverage of MCH care, many countries are lagging behind on MDG 4 and 5, in particular with respect to reducing neonatal and maternal mortality [1]. This has much to do with the difficulties of increasing the rate of institutional deliveries, especially among the poorest population groups within countries. Possible barriers for using delivery care can be financial, cultural, or knowledge related. Both demand side programs such as conditional cash transfers, vouchers and user fee removals, and supply side programs such as performance based financing, are increasingly being implemented to increase the coverage of MCH care [2–5].

In India, the bulk of health care is still financed by out-of-pocket payments made at the point of health care use, leading to problems of inaccessibility and lack of financial protection [5]. In India, progress towards MDG4 and 5 has been modest; neonatal mortality has fallen from 44 per 1000 live births in 2001 to 38 per 1000 live births in 2005 and 28 per 1000 live births in 2015. Maternal mortality is still high with 280 per 100,000 and 174 per 100,000 live births in 2005 and 2015, respectively [6]. Data from the latest Demographic Health Survey (2005) indicates that only 40% of women deliver in an institution [7]. Socioeconomic inequalities in MCH have remained high in the past decade [8].

With the aim of reducing neonatal and maternal mortality and reducing out-of-pocket payments associated with institutional delivery, the Indian Government introduced the Janani Suraksha Yojana (JSY) scheme in 2005. JSY is a conditional cash transfer program that financially rewards pregnant women for delivery, and especially for delivery in a facility that is empanelled by JSY. Even though the scheme is centrally sponsored, eligibility criteria vary by state. The poorest (low performing) states target all women, while in high performing (less poor) states, only women holding a Below Poverty Line (BPL) card are eligibility. Some states require that delivery takes place in a public facility, while other states (such as Odisha) also have accredited private providers [9]. Hospital delivery in a government facility should in theory be free of charge. Free transport to facility should also be available for pregnant women and newborns in both states. It is likely however that these services are not always free in practice, and there may still be other costs associated with delivery in a facility. In Jharkhand, during the study period, women also received a small amount of money for home deliveries.

With JSY being one of the largest conditional cash transfer programs worldwide [5], a considerable number of studies have sought to establish its impact on the proportion of women delivering in a facility. Most notably, Lim et al. (2010) found JSY to have a large positive impact on the institutional delivery rate (an increase of 43–49% in the probability of women delivering in a facility), leading to important reductions in neonatal mortality (a reduction of 2.3 neonatal deaths per 1,000 live births) [10]. These large effects have, however, been questioned by Powell-Jackson et al. (2015) and Joshi and Sivaram (2014) who suggested that, despite an increase in use of maternity health services, there was no effect of JSY on maternal and neonatal health due to the low quality of care provided in public facilities [11, 12]. Some studies investigating heterogeneity of the effect of JSY on use of maternity care across socioeconomic groups found that poorer, less educated women and those belonging to Scheduled Castes or Tribes (as defined by the Indian Constitution) generally benefit more from JSY [11, 13, 14]. Yet, these studies do not describe and explain socioeconomic inequalities in JSY receipt in detail.

In this study, we aimed to measure the magnitude of socioeconomic inequality in the receipt of JSY benefits and explain these inequalities using the decomposition method. We use data that was prospectively collected through a demographic surveillance system in the states of Jharkhand and Odisha.

## Methods

### Data

We used data from a population surveillance system in five districts in the states of Jharkhand and Odisha, two of the poorest states in India (Godda, Khunti, and Ranchi district in Jharkhand, and Mayurbhanj and Rayagada district in Odisha). Data were only collected in three out of 24 districts in Jharkhand and in two out of 30 districts in Odisha. The surveillance system was set up as part of a scale-up of a community-based women’s group intervention to reduce neonatal mortality and improve maternal and neonatal health. The intervention had proven to be successful in a cluster randomised trial in Jharkhand and Odisha, and had been scaled up to five districts in these states that were not part of the original trial. The ‘scale-up area’ was divided into an intervention area in which women’s groups were set up, and a control area, in which no women’s groups were initially set up (at a later stage, this control area for the scale-up became the site of a new randomised trial with women’s groups facilitated by government community health workers (ASHAs)) [15]. We analysed the data for the control arm of the scale-up area, including births in the period 1 January 2009 (when the surveillance system was set up) until 31 August 2010 (after which women’s groups were set up in the control area as part of the new trial). The control area consisted of 25 clusters in five districts, with a total population of around 35,000 (around 7000 per district). Of the study population, 20,000 were intensively monitored, collecting information on vital outcomes and secondary outcomes including receipt of JSY benefits. For the remaining 15,000 population, only data on vital outcomes and a limited set of secondary outcomes, not including JSY uptake, were collected. We analysed the data for the population that was intensively monitored, which gave us a sample of 3682 births.

The main outcome variable of interest reflects whether or not the woman received JSY benefits for her delivery during the study period (1/0) representing the uptake of JSY. Other outcomes include the uptake of at least three antenatal care visits (ANC3) and delivery in a public facility^1^.

Our main socioeconomic variable of interest is economic status, using a wealth index derived from principal component analysis on a range of asset variables as proxy^2^. Second, we have an indicator for whether the infant’s mother belongs to a Scheduled Tribe or Scheduled Caste (ST/SC), who represent the most socioeconomically disadvantaged groups, recognized by the Constitution of India [16, 17]. Third, we define an indicator for illiteracy reflecting whether or not the infant’s mother can read and write (1/0), as verified by the interviewer.

Other covariates include demographics (age, number of previous pregnancies) and geographical characteristics (district indicators).

### Measuring socioeconomic inequality

Next to summarizing JSY uptake across wealth quintiles, we also measured socioeconomic inequality in the uptake of JSY using a concentration index, which takes into account inequality across the full socioeconomic distribution. Erreygers [18] has argued that the standard concentration index has some shortcomings when applied to bounded variables, most importantly that the bounds of the index are dependent of the mean of the indicator. We therefore applied the corrected concentration index (${CC}_y$ ) which, for our binary outcome of JSY receipt (${y}_i\Big)$ , is calculated as:

1 $$ C{C}_y=8 c o v\left({y}_i\cdot {R}_i\right) $$

where ${R}_i$ reflects a woman i’s fractional rank in the socioeconomic distribution. This corrected index shares the same interpretation as the standard concentration index. Negative values imply that JSY uptake is more concentrated among poorer women. If all women, irrespective of their socioeconomic status, are equally likely to receive JSY benefits, the index would equal to zero, and (iii) transferring JSY uptake from a richer to a poorer woman reduces the value of the index [18].

### Determinants of JSY uptake

To identify the important determinants of JSY uptake, we ran several linear probability models^3^. First, we ran a model of JSY uptake (*y*_*i*_ ) only on the wealth quintile indicators (model 1). Next we added household/mother covariates and district indicators (model 2). Thereafter we added an indicator for whether the child was born in a public facility – one of the most important conditions for eligibility for JSY benefits (model 3). Finally we estimated model 2 only on the sample of births that took place in a government facility (model 4).

### Decomposition of socioeconomic inequalities in JSY uptake

Having established the determinants of JSY uptake, we wanted to identify the most important drivers of socioeconomic inequality in JSY uptake. If we assume that JSY uptake can be written as a linear function of K determinants ${x}_k$,

2 $$ {y}_i=\alpha +{\displaystyle {\sum}_{k=1}^K{\beta}_k{x}_{i k}+{\upvarepsilon}_i}, $$

the corrected concentration index can be written as a weighted average of the concentration indices of the covariates (${C}_{x_k}\Big)$ :

3 $$ C{C}_y=4\left[{\displaystyle {\sum}_{k=1}^K{\beta}_k{\overline{x}}_k}{C}_{x_k}+ G{C}_{\varepsilon}\right] $$

Where ${\overline{x}}_k$ is the mean of x, and ${GC}_{\varepsilon}$ is the generalized concentration index of the residuals. Equation (3) illustrates that for a covariate to contribute to socioeconomic inequality in JSY uptake it needs to be associated with JSY uptake (${\beta}_k\Big)$ and be unequally distributed across socioeconomic status (${C}_{x_k}\Big)$ [18, 19].

These decompositions were performed for models (2), (3) and (4) as introduced in the previous section. All analyses were performed in Stata 12. Standard errors are adjusted for clustering on the primary sampling unit.

## Results

### Summary statistics

**Fig. 1:**
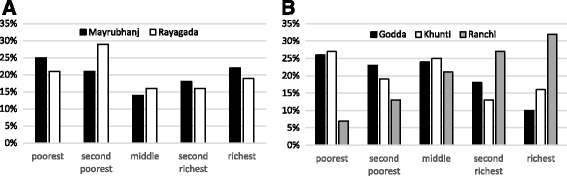
Distribution of household wealth across districts in Odisha (**a**) and Jharkhand (**b**). Notes: Figures show the proportion of children in each wealth quintile in each district, by state

Table 1 shows the means of our covariates. The characteristics of women were largely similar in the two states, except for a higher percentage of women from Scheduled Castes or Scheduled Tribes in Odisha (86%) than in Jharkhand (62%). In Jharkhand, there were considerable district-level differences in the characteristics of the study participants. The percentage of women from Scheduled Casts or Scheduled Tribes varied from 49% in Godda to 91% in Kunthi, and the number of previous pregnancies varied from 1.4 in Ranchi to 1.9 in Khunti. Between-district differences in literacy rate were large in both states. Poor-rich differences in the above characteristics were also large in both states. Richer women had on average 1 previous pregnancy, compared with 2 pregnancies among poorer women. Furthermore, the illiteracy rate and percentage belonging to a Scheduled Caste or Scheduled Tribe was substantially lower among the richest quintile in both states. Differences in household wealth between districts were especially large in Jharkhand, and much smaller in Odisha (Fig. 1a and b).

**Table 1:** Summary statistics of covariates and distribution across districts and socioeconomic status

	Age (years)		Number of previous pregnancies		Scheduled tribe / scheduled caste (%)		Illiteracy rate (%)	
	Odisha	Jharkhand	Odisha	Jharkhand	Odisha	Jharkhand	Odisha	Jharkhand
Total population	24.9	24.9	1.8	1.6	86	62	69	69
By district								
Mayurbhanj	23.9		1.7		91		56	
Rayagada	26.1		1.9		81		83	
Godda		23.9		1.6		49		78
Khunti		26.4		1.9		91		77
Ranchi		24.9		1.4		56		54
By wealth group								
Poorest quintile	25.5	25.1	2.4	1.8	93	68	87	90
Second poorest quintile	25.5	25.4	2.1	1.9	88	70	82	88
Middle quintile	24.5	25.4	1.6	1.8	92	69	78	77
Second richest quintile	24.4	24.6	1.4	1.5	89	57	63	63
Richest quintile	24.2	23.7	1.1	1.1	68	44	30	29
Concentration index (SD)	−0.01 (0.00)	−0.01 (0.00)	−0.16 (0.02)	−0.10 (0.01)	−0.17 (0.02)	−0.21 (0.02)	−0.45 (0.03)	−0.49 (0.02)

Notes: Table shows the standard concentration index for continuous variables (age, number of previous pregnancies) and the corrected concentration index for bounded variables (scheduled tribe/scheduled caste, illiteracy rate)

Table 2 shows average awareness and receipt of JSY benefits across the two states and districts within each state, and across wealth quintiles within each state. The vast majority of women had heard of the scheme (94% in Odisha, 85% in Jharkhand). There was some district-level variation in Odisha, where only 62% of women in Khunti had heard of JSY, compared with 97% in Ranchi. While poor-rich inequalities in awareness were small in Odisha, there was some pro-rich inequality in Jharkhand (CI = 0.18, SD 0.02).

**Table 2:** Summary statistics of JSY-related outcomes and their distribution

	Heard (%)		Received (%)		Total amount (Rs.)	
	Odisha	Jharkhand	Odisha	Jharkhand	Odisha	Jharkhand
Total population	94	85	62	20	1402	1222
By district						
Mayurbhanj	95		59		1405	
Rayagada	93		66		1400	
Godda		87		5		944
Khunti		62		13		808
Ranchi		97		40		1343
By wealth group						
Poorest quintile	91	71	56	11	1398	1015
Second poorest quintile	95	81	62	16	1394	1101
Middle quintile	94	86	59	15	1392	1290
Second richest quintile	96	92	64	25	1418	1256
Richest quintile	96	92	71	33	1408	1280
Concentration index (SD)	0.04 (0.02)	0.18 (0.02)	0.10 (0.03)	0.18 (0.02)	0.00 (0.00)	0.04 (0.01)
Total sample (N)	1345	2337	1345	2337	836	470

Notes: Table shows the standard concentration index for continuous variables (amount) and the corrected concentration index for bounded variables (having heard about JSY, having received JSY benefits). Total amount received in Indian Rupee (Rs.) was estimated among women who received JSY benefits

The percentage of women that received JSY benefits was low in comparison with the percentage of women that had heard of the scheme, especially in Jharkhand. While 62% of women received the benefits in Odisha, only 20% did so in Jharkhand. In the latter state, uptake of JSY benefits ranged from 5% in Godda to 40% in Ranchi, while such regional differences were small in Odisha. In both states, the receipt of JSY benefits was disproportionally concentrated among better-off women (CI: 0.18 in Jharkhand, 0.10 in Odisha).

Women who took up the benefits, received about 1400 Rs. on average, but reported amounts were somewhat lower in Jharkhand (1222 Rs.). There was little socioeconomic inequality in the amount received, although in Jharkhand women in the lowest quintile reported receiving on average 265 Rs. less than those in the highest wealth quintile. These lower amounts for Jharkhand are arguably explained by the higher proportion of home births (4% in Jharkhand versus 2% in Odisha) and the small benefits associated with home deliveries.

The above patterns of inequality in the receipt of JSY benefits reflect inequalities in uptake of maternity care (Table 3). In Jharkhand, the overall uptake of maternity care was lower, and inequalities between districts and wealth groups were larger, than in Odisha. In Jharkhand, only 26% of women made at least 3 antenatal care visits and 27% of women delivered in a facility. While district level differences were small in Odisha, they were large in in Jharkhand (8–55% for deliveries in government facilities and 17–44% for 3ANC). In both states, the majority of institutional deliveries took place in a public facility. The percentage of private sector deliveries was only substantial for the richest quintile, especially in Jharkhand.

**Table 3:** Summary statistics of maternity care-related outcomes and their distribution

	ANC3 (%)		Institutional delivery (%)					
	Odisha	Jharkhand	All		Public		Private	
			Odisha	Jharkhand	Odisha	Jharkhand	Odisha	Jharkhand
Total population	49	26	73	27	64	20	9	7
By district								
Mayurbhanj	44		75		74		2	
Rayagada	54		71		53		17	
Godda		10		17		12		5
Khunti		8		17		12		5
Ranchi		55		44		34		10
By wealth group								
Poorest quintile	36	11	65	17	60	13	6	4
Second poorest quintile	43	15	70	17	60	14	9	3
Middle quintile	49	15	68	17	62	14	6	3
Second richest quintile	52	33	76	32	67	27	9	5
Richest quintile	67	57	87	53	73	34	14	20
Concentration index (SD)	0.25 (0.03)	0.37 (0.02)	0.16 (0.03)	0.30 (0.02)	0.11 (0.03)	0.19 (0.02)	0.05 (0.02)	0.11 (0.01)
Total sample (N)	1345	2337	1345	2337	1345	2337	1345	2337

Notes: Table shows the corrected concentration index

### Determinants of JSY uptake

Table 4 presents the regression results of the different models used to identify the determinants of JSY uptake. Model (1) confirms the earlier described patterns of pro-rich receipt of JSY benefits. These poor-rich inequalities were largely explained by district-level differences in JSY uptake and by characteristics of the mother (Model 2). In Jharkhand, the probability of receiving JSY benefits was highest in Ranchi (33 percentage points (PP) higher than in the reference district Godda) and five PP lower for illiterate women than for literate women. In Odisha, there were no statistically significant district level differences in JSY receipt, but women who had had more previous pregnancies were less likely to receive JSY benefits (4 PP).

**Table 4:** Determinants of JSY uptake

	Odisha				Jharkhand			
	[1]	[2]	[3]	[4]	[1]	[2]	[3]	[4]
Household wealth								
Poorest (ref.)								
2nd poorest	0.06^b^	0.04	0.02	0.01	0.04^b^	0.00	0.01	−0.03
Middle	0.03	−0.00	−0.01	−0.01	0.04	−0.03	−0.02	−0.00
2nd richest	0.08^a^	0.04	0.02	−0.02	0.14^a^	0.01	−0.01	−0.01
Richest	0.15^c^	0.09	0.04	−0.05	0.22^c^	0.04^a^	0.01	−0.06
Region								
Mayurbhanj (ref.)								
Rayagada		0.07	0.21^c^	0.14^b^				
Godda (ref.)								
Khunti						0.08	0.06^a^	0.27^c^
Ranchi						0.33^c^	0.26^c^	0.48^c^
Age		0.01	0.00	0.01		−0.00	−0.00	−0.00
Previous pregnancy		−0.04^b^	−0.01	−0.00		0.00	0.00	0.02
ST/SC		−0.02	0.02	−0.02		0.01	0.02^a^	−0.03
Illiterate		−0.03	−0.03	−0.02		−0.05^b^	−0.03^a^	−0.04
Public-institutional delivery			0.64^c^				0.37^c^	
Observations	1345	1344	1342	860	2335	2329	2315	469

Note: Table shows coefficients from linear probability models with JSY uptake as dependent variable. The fourth [4] model is only estimated on the sample of women who delivered in a public facility

^a^significant at 10% level, ^b^significant at 5% level, ^c^significant at 1% level

In Model 3, we added an indicator for government facility delivery to the covariates. As delivery in a facility was a condition for receiving JSY benefits, and there are few private hospitals empanelled in JSY. Delivery in government facility was strongly associated with JSY receipt in both states, although more so in Odisha (64 PP, versus 37 PP in Jharkhand). In this model, poor-rich inequalities in JSY receipt disappeared, but there still was substantial district level heterogeneity. In Odisha, women from Rayagada were 21 PP more likely to receive JSY benefits than women from Mayurbhanj; in Jharkhand, women from Ranchi were 26 PP more likely to receive JSY benefits than women from Godda. Model (4) is similar to model (2) but only included births in government facilities. Within this sample, the probability of receiving JSY benefits only varied by district.

### Decomposition of socioeconomic inequality in JSY uptake

**Fig. 2:**
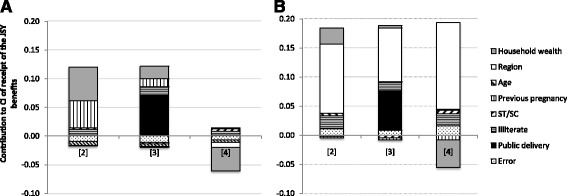
Decomposition of socioeconomic inequality in JSY uptake in Odisha (**a**) and Jharkhand (**b**). Notes: The bars in each figure reflect absolute contributions to inequality in JSY uptake using model [2], [3] and [4] in Table 4, respectively. In Odisha, CI of JSY uptake equals 0.10 the full sample (model 2 and 3), and −0.05 for the sample of public deliveries (model 4). In Jharkhand, CI equals 0.18 and 0.14, respectively

Figure 2a and b graphically illustrate the results of the decomposition of poor-rich inequality in JSY receipt as measured by the concentration index, for models (2), (3) and (4) in Table 4 (detailed results are available in the Appendix). The total height of the bar represents the magnitude of poor-rich inequalities (CI of JSY receipt). Note that a variable contributes to the CI both through its association with JSY receipt (Table 4) and the extent to which it is unequally distributed by household wealth (Table 1). As model (2) and model (3) were estimated on the same sample, the bars are equally high. The decomposition using model (4) only included births in a government facility.

Looking at Fig. 2a for Odisha, we see in the first bar that household wealth was the most important driver (43%) of poor-rich inequality in JSY uptake; the second most important driver was the number of previous pregnancies (34%). The latter contribution was driven by the higher number of previous pregnancies among poorer women and the negative association between the number of pregnancies and JSY uptake. These contributions reduced substantially after adding government facility delivery to the model (second bar). Within the sample of government facility deliveries (third bar), receipt of JSY benefits was pro-poor (CI = −0.05, SD 0.03); poor women were slightly more likely to obtain JSY benefits than richer women.

In Jharkhand (Fig. 2b), we get quite a different story, with district level differences explaining most of the poor-rich inequalities in each of the decomposition models (63, 47 and 60% in models (2), (3) and (4) respectively). Even within the sample of government facility deliveries, there was considerable pro-rich inequality in JSY receipt (CI = 0.14 SD 0.05), which was also driven by district level differences. This can be explained by the fact that in Ranchi, the richest district, women were far more likely to obtain JSY benefits than in the other districts. Once these district level differences were taken into account, JSY uptake was more concentrated among the poor, which explains the negative contribution of wealth to the poor-rich inequalities in JSY uptake (−19%).

## Discussion

Our analysis shows that inequalities in JSY receipt are substantial in Jharkhand and Odisha, both between wealth groups and between districts within states. In Jharkhand, these inequalities to some extent reflected differences in awareness of the scheme. Nevertheless, the discrepancy between awareness and receipt of benefits was very large in both states, especially in Jharkhand. Poor-rich inequalities in JSY receipt reflected substantial pro-rich inequalities in the institutional delivery rate. Yet, even among government facility deliveries there were considerable pro-rich inequalities in JSY receipt in Jharkhand, which were explained by district level differences in poverty and receipt of JSY benefits. Conversely, in Odisha, poorer women delivering in a government institution were at least as likely to receive JSY benefits as richer women.

There are some limitations to this study. First, the data were not collected with the aim of evaluating JSY, so we could not exactly identify whether women had fulfilled all the necessary conditions to be eligible for JSY benefits. We used delivery in a government facility as a proxy, as this is the most important requirement. Second, the data were only collected in a small number of districts in both states. The large district level inequalities in Jharkhand may reflect the fact that data were collected in Ranchi district – the capital district- and two rural districts, while in Odisha the capital city district was not included. Third, our data relates to births in 2009 and 2010. While the implementation of the JSY program may have improved in the mean-time, our paper provides one of the most recent estimates available for JSY uptake across socioeconomic strata. Fourth, given the cross sectional nature of our data, our models only allow the identification of associations between covariates and JSY uptake, and should not be interpreted as causal effects.

Our findings correspond with DLHS evidence that the odds of receiving JSY benefits were not always highest among the poor. Yet, the poor-rich inequalities in JSY receipt that we report for Odisha and Jharkhand were much larger than in these national level studies [5, 10].

Our finding of large between-state differences in JSY uptake reflects a broader pattern of a highly variable implementation process and substantial differences in JSY uptake across states in the country. Interestingly, Odisha was the next-highest performing state in terms of JSY receipt in the country, and Jharkhand one of the lowest performing states, based on 2007–2009 DLHS data. We found that even within states, indicators of JSY success were highly variable between districts. Such differences could be due to differences in accessible health infrastructure for facility delivery, but could also highlight differences in state-level government capacity to implement national level policies [10].

### Implications

Our findings imply that the JSY scheme is currently not sufficient to close the poor-rich gap in institutional delivery rate. Low uptake is not so much related to low awareness of the scheme per se, but rather with remaining barriers to institutional delivery and, in Jharkhand, to district-level differences in performance of the JSY scheme. This corresponds with findings from another study, which reported that despite receiving JSY benefits, many families still have to borrow money to cover out of pocket expenditures. Furthermore, non-monetary demand and supply side barriers, including quality of care, distance, and a tradition of home delivery remain [5]. The large differences in receipt of JSY and uptake of maternity care between Jharkhand and Odisha and between districts within Jharkhand, suggest that more support is needed for low performing districts and states. Women giving birth in government facilities in Ranchi, the richest district, were much more likely to receive benefits as compared to those giving birth in government facilities in other districts. This might reflect a better administration of the scheme in districts with more resources. It is reassuring, though, that we found that poor women were as likely to receive JSY benefits as richer women when delivering in a government facility, after taking such district-level differences into account.

Our findings are in line with results from other evidence looking at the equity in the uptake of cash incentive schemes in neighboring countries of India. A review of the evidence has revealed that the Safe Delivery Incentive Programme in Nepal and the Maternal Health Voucher scheme in Bangladesh failed to target poorer households. The uptake of those schemes was more concentrated among the better-off [20].

## Conclusions

JSY benefits were not equally distributed, favouring wealthier groups. These inequalities in turn reflected pro-rich inequalities in the institutional delivery. The JSY scheme is currently not sufficient to close the poor-rich gap in institutional delivery rate. Important barriers to institutional delivery remain to be addressed and more support is needed for low performing district and states.

## Appendix

**Table 5:** Decomposition of socioeconomic inequality in JSY uptake in Odisha and Jharkhand

	Odisha (%)			Jharkhand (%)		
	[2]	[3]	[4]	[2]	[3]	[4]
Household wealth	42.78	15.05	−55.01	14.68	2.14	−19.10
Region	−0.30	−0.84	−11.50	63.17	46.92	59.83
Age	−5.59	−2.96	−5.79	2.39	0.37	0.63
Previous pregnancy	34.34	10.31	2.91	−1.28	−1.47	−3.10
ST/SC	2.01	−1.86	5.36	−0.86	−2.41	2.51
Illiterate	8.47	10.64	10.95	11.89	7.56	8.23
Public-insitutional delivery	0.00	50.27	0.00	0.00	34.95	0.00
Error	−6.51	−8.07	−8.48	5.72	4.18	6.60

In Jharkhand, women received JSY payments in installments. However, women received 500 Rs. for home delivery event. This was continued during the data collection period. Number of home delivery might have some influence in reducing the average

## Footnotes

1 In Jharkhand, only deliveries in public facilities were eligible for JSY benefits, while in Odisha also some private facilities were included in the JSY scheme. Unfortunately we do not know whether the private facilities reported to have been used in our data are accredited by JSY. Therefore, we cannot identify exactly whether women have fulfilled all requirements to be eligible for JSY benefits. In Jharkhand, where only births in government facilities are eligible for JSY, we only found 13 births outside of government facilities that were given the cash transfer of 1400 Rs. This does not suggest major problems of leakage of the program.

2 Assets include: mattress, bed, chair, table, pressure cooker, electricity, fan, radio, television, clock, phone, animal-drawn cart, bicycle, motor bicycle and agricultural land ownership (small size, medium size, large size and mortgage).

3 We have confirmed robustness of results to using probit models and estimating marginal effects. We prefer to present the linear probability model as this facilitates the decomposition technique that is used later. Non-linear extensions of the decomposition method do exist but require focusing on the latent index rather than the actual uptake variable or impose approximation errors (Vandoorslaer, Koolman and Jones 2004) [21].
